# Orbit constraint of a small bead on a rotating large circular hoop in a horizontal plane

**DOI:** 10.1038/s41598-024-53713-w

**Published:** 2024-03-08

**Authors:** Yuan-Sheng Wang

**Affiliations:** College of Science, Hunan University of Science and Engineering, No. 130, Yangzitang Road, Lingling District, Yongzhou, 425199 Hunan China

**Keywords:** Constraint force, Rotating hoop, Differential equation of motion, Friction, Energy science and technology, Engineering, Physics

## Abstract

Orbit constraint problems can be encountered in mechanical equipment and amusement equipment. Mechanics exercises generally consider the ideal physical model, and the practical problems also consider the influence of friction, which makes the problem more complex and and practical. The problem of the force and oscillation of objects on orbit needs to be deeply discussed. In order to simulate the orbital motion of objects more realistically and help students expand their theoretical mechanics beyond class, we study the orbit constraint of a small bead on a rotating large circular hoop in a horizontal plane about an axis passing through a point on the circumference. The coupling equations followed by the bead on the hoop are derived using Newton’s second law in a planar polar coordinate system and solved by numerical methods. We found that under the action of friction, when the initial angular velocity of the bead is greater than the critical angular velocity, the bead will rotate on the ring, and the number of rotations is related to the initial angular velocity and influenced by the friction coefficient. At different initial angular velocities, the number of oscillations of the bead on the hoop is basically the same and ultimately stops near the fixed point.

## Introduction

Orbit constraint problems can be encountered in mechanical and entertainment equipment. It is also an important issue in theoretical mechanics. When a bead or ring is regarded as a particle moves on a curved orbit, it will be subject to the constraint force of the orbit. In mechanics textbooks, only ideal models are generally considered, and the practical problems also consider the influence of friction, which makes the problem more complex and practical. In order to simulate the orbital motion of objects more realistically and help students expand their theoretical mechanics beyond class, the problem of the force and oscillation of objects on orbit needs to be deeply discussed. Due to the general curvilinear motion of objects, natural coordinate systems are often used. When solving the constraint problem, the constraint is generally removed, replaced by the constraint force, and the moving object is regarded as a free particle. The constraint force is generally unknown, unlike ordinary forces. It is not entirely determined by the constraint itself, but is related to other forces acting on the particle and the motion state of the particle itself. Moreover, relying solely on the constraint force itself cannot cause any movement of the particle, so the constraint force is often referred to as the passive force. The constraint force usually acts on the contact point between a particle and a curve or surface. In the absence of friction, it follows the normal of a curve or surface, while in the presence of friction, it slopes at a certain angle to the normal^1^.

The motion of a bead on a circular hoop is one of the orbital constraint problems. The movement of the bead exhibits various motion modes, such as oscillation on one side of the hoop and rotation around the hoop. It also displays a series of characteristics of dynamic systems, such as fixed points, bifurcation, reversibility, symmetry breaking, etc^2–7^. In theory, Johnson et al. studied a bead on a hoop rotating about a horizontal axis and developed a new approach for creating a one-dimensional, gravitational ponderomotive trap^5^. Dutta et al. explored the diverse modes of motion of a bead moving on a vertically rotating circular hoop, including frictionless and frictional motion^6^. Animasaun et al. pointed out that a critical function of starting angular velocity is calculating the overall angular displacement of a rotating object^8^. We previously studied the constraint problem of a small ring on an elliptical orbit, and analyzed the influence mechanism of velocity and orbit shape on the constraint force and motion of the ring^9^.

For a smooth orbit, it is possible to obtain the analytical result of the orbital motion equation of the particle, but when considering friction, the actual situation is much more complex, and then it needs to be solved by numerical method. In this paper, the orbit constraint of a bead on a rotating large circular hoop in a horizontal plane about an axis passing through a point on the circumference is studied. We need to solve a second-order differential equation, which can be transformed into a first-order coupled system of equations by reducing the order. We can solve it numerically using the forward difference formula. Because the calculation time is relatively short, the time step can be taken very small, which ensures the calculation accuracy. Because one of the equations is complex, it is not easy to obtain its numerical solution. Fortunately, we can regard it as a quadratic equation about the angular velocity of the next step time, and the roots of the angular velocity can be obtained by using the root formula. Its two roots correspond to the clockwise and counterclockwise rotation of the bead relative to the hoop.

By solving the above equations, this paper mainly studies three aspects of problems. Firstly, the oscillation of the bead on the hoop without friction is studied. Secondly, the influence mechanism of friction coefficient on the motion of the bead under a given initial angular velocity is studied. Thirdly, the influence mechanism of initial angular velocity on the motion of the bead under the action of friction is studied. By solving the eigenvalues of the Jacobian matrix of the system, we analyzed the fixed point types of the phase diagram, and the conclusion is consistent with the numerical results. We hope these studies can provide some references for similar amusement facilities and engineering design.

## Model equations

**Figure 1:**
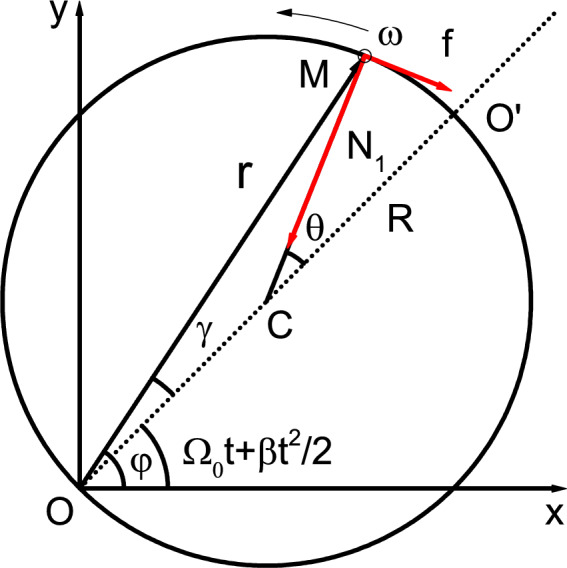
A schematic diagram of force analysis of a bead on a rotating circular hoop with radius *R* when the angular velocity $\omega >0$ of the bead relative to hoop. $\Omega _0 t+\beta t^2/2$ is the rotation angle of the $OO^{'}$ axis of the rotating hoop relative to the *x* axis, $\gamma$ is the included angle between *MO* and $OO^{'}$, and $\varphi =\Omega _0 t+\beta t^2/2+\gamma$. $\theta$ is the rotation angle of the bead relative to the hoop. $N_1$ and *f* are the constraint force and friction of the bead, respectively.

We consider the motion of a bead that can be regarded as a particle on a rotating large hoop. As shown in Fig. 1, there is a large circular hoop with radius *R* in the horizontal plane. A bead with mass *m* is set at point *M* on the hoop, and the friction coefficient between it and the hoop is $\mu$. Let the hoop rotate around a point *O* on the hoop at an angular velocity $\Omega =\Omega _0+\beta t$ in the horizontal plane, where $\Omega _0$ is the initial angular velocity and $\beta$ is the angular acceleration. The plane cartesian coordinate system $O-xy$ and the polar coordinate system are established, with *O* as the pole and *x* axis as the polar axis. Let the center of the hoop be *C*, connect *OC* and extend it, and its included angles with *MO* and *MC* are $\gamma$ and $\theta$ respectively. We specify that the counterclockwise rotation direction is positive, so the position of the bead on the hoop can be described by $\theta$. We set the initial angular velocity of the bead relative to the hoop as $\omega =\omega _0$. Figure 1 shows the force analysis of the bead when the angular velocity $\omega >0$, where $N_1$ is the constraint force pointing to the center of the hoop, and its normal and angular components are $N_r$ and $N_\varphi$, respectively, *f* is the friction, and its normal and angular components are $f_r$ and $f_\varphi$, respectively. In addition, the bead is also subjected to gravity *mg* and vertical supporting force $N_2$, where *g* is gravity acceleration, $N_2=mg$ and the friction $f=\mu \sqrt{N_1^2+N_2^2}$. The constraint force here forces the bead to make curvilinear motion on the hoop, and its size depends on the gravity, angular velocity and angular acceleration of the bead. Friction hinders the relative movement of the bead and the hoop. The coordinates of the bead in polar coordinates satisfy the relationship $r=2R\cos \gamma$, $\varphi =\varphi _0+\Omega _0 t+\beta t^2/2+\gamma$, where $\varphi _0$ is initial angle, and $\varphi _0=0$ when $OO^{'}$ and *x* axes coincide.

The differential equation of motion of the bead varies according to the value range of $\theta$. Let $\theta _0=\textrm{mod}(\theta ,2\pi )$ denote the remainder of $\theta /2\pi$, that is, $\theta =2n\pi +\theta _0 (n=0,1,2,3\cdots )$. When $\theta _0\in (0,\pi )$ or $(-\pi ,0)$, $\gamma =\theta _0/2$. When $n=2k$ and $n=2k+1 (k=0,1,2,3\cdots )$, the differential equations of motion of the bead are the following Eqs. (1) and (2), respectively^1,2,10^.

1a $$\begin{aligned} m\left( \ddot{r}-r\dot{\varphi }^2\right)&=-N_1\cos \frac{\theta }{2}{\pm }f\sin \frac{\theta }{2}, \end{aligned}$$

1b $$\begin{aligned} m\left( r\ddot{\varphi }+2\dot{r} \dot{\varphi }\right)&=-N_1\sin \frac{\theta }{2}{\mp }f\cos \frac{\theta }{2}. \end{aligned}$$

2a $$\begin{aligned} m\left( \ddot{r}-r\dot{\varphi }^2\right)&=N_1\cos \frac{\theta }{2}{\mp }f\sin \frac{\theta }{2}, \end{aligned}$$

2b $$\begin{aligned} m\left( r\ddot{\varphi }+2\dot{r} \dot{\varphi }\right)&=N_1\sin \frac{\theta }{2}{\pm }f\cos \frac{\theta }{2}. \end{aligned}$$

When $\theta _0\in (\pi ,2\pi )$, $\gamma =(\theta _0-2\pi )/2$. When $\theta _0\in (-2\pi ,-\pi )$, $\gamma =(\theta _0+2\pi )/2$. When $n=2k+1$ and $n=2k$, the equations are the same as Eqs. (1) and (2), respectively. In the Eqs. (1) and (2), the symbols ${\pm }$ and ${\mp }$ from top to bottom represent counterclockwise $\omega >0$ and clockwise $\omega <0$ respectively.

The coupled equations about $\theta$ and $\omega$ can be derived by using Eqs. (1) and (2).

3a $$\begin{aligned}&{\dot{\omega }}^2+2Q\left( \theta ,t\right) {\dot{\omega }}+Q^2\left( \theta ,t\right) -\frac{\mu ^2g^2}{R^2}-\mu ^2\Big ({\omega ^2}+2\omega \left( \Omega _0+\beta t\right) \nonumber \\&\quad {+2\left( \Omega _0+\beta t\right) ^2\cos ^2\frac{\theta }{2}-\beta \sin \theta }\Big )^2=0, \end{aligned}$$

3b $$\begin{aligned}&\omega =\dot{\theta }, \end{aligned}$$

where $Q\left( \theta ,t\right) =2\beta \cos ^2({\theta }/{2})+\left( \Omega _0+\beta t\right) ^2\sin \theta$. We use forward difference formula to solve the above equations, and the difference schemes are as follows

4a $$\begin{aligned}&{\omega _{i+1}}^2+2\Delta t\left( Q\left( \theta _i,t_i\right) -\frac{\omega _i}{\Delta t_i}\right) \omega _{i+1}+{\omega _i}^2-2Q\left( \theta _i,t_i\right) \omega _i\Delta t\nonumber \\&\quad +Q^2\left( \theta _i,t_i\right) {\Delta t}^2-\frac{\mu ^2g^2{\Delta t}^2}{R^2}-\mu ^2{\Delta t}^2\Big ({{\omega _i}^2+2\left( \Omega _0+\beta t_i\right) ^2\cos ^2\frac{\theta _i}{2}}\nonumber \\&\quad {+2\omega _i\left( \Omega _0+\beta t_i\right) -\beta \sin \theta _i}\Big )^2=0, \end{aligned}$$

4b $$\begin{aligned}&\omega _i=\frac{\theta _{i+1}-\theta _i}{\Delta t}. \end{aligned}$$

Equation (4a) can be regarded as a univariate quadratic equation about $\omega _{i+1}$.

5 $$\begin{aligned} {\omega _{i+1}}^2+b_i\omega _{i+1}+c_i=0, \end{aligned}$$

where $b_i=2\Delta t\left( Q\left( \theta _i,t_i\right) -\omega _i/\Delta t_i\right)$, $c_i={\omega _i}^2-2Q\left( \theta _i,t_i\right) \omega _i\Delta t+Q^2\left( \theta _i,t_i\right) {\Delta t}^2 -{\mu ^2g^2{\Delta t}^2}/{R^2}-\mu ^2{\Delta t}^2({\omega _i}^2+2\left( \Omega _0+\beta t_i\right) ^2\cos ^2({\theta _i}/{2})+2\omega _i\left( \Omega _0+\beta t_i\right) -\beta\sin \theta _i)^2$. It has two roots $\omega _{i+1}=({-b_i{\pm }\sqrt{b_i^2-4c_i}})/{2}$. The $+$ and − in symbol ${\pm }$ correspond to the clockwise and counterclockwise rotation of the bead relative to the hoop, respectively. According to Taylor’s formula, the difference schemes have a first-order accuracy of $O(\Delta t)$.

From Eqs. (1) and (2), the expression of the constraint force $N_1$ can be deduced as (e.g., eliminating *f* with Eq. (1a)$\times \cos \frac{\theta }{2}+$ Eq. (1b)$\times \sin \frac{\theta }{2}$ yields $N_1$).

6 $$\begin{aligned} N_1=mR\left( {\dot{\theta }}^2+2\left( \Omega _0+\beta t\right) ^2\cos ^2\frac{\theta }{2}+2\dot{\theta }\left( \Omega _0+\beta t\right) -\beta \sin \theta \right) . \end{aligned}$$

The radial and angular components of the constraint force $N_1$ and friction *f* are $N_r$, $N_\varphi$, $f_r$ and $f_\varphi$ respectively.

7 $$\begin{aligned} N_r= & {} {\pm } N_1\cos \frac{\theta }{2}, \end{aligned}$$

8 $$\begin{aligned} N_\varphi= & {} {\pm } N_1\sin \frac{\theta }{2}, \end{aligned}$$

9 $$\begin{aligned} f_r= & {} {\pm } f\sin \frac{\theta }{2}, \end{aligned}$$

10 $$\begin{aligned} f_\varphi= & {} {\pm } f\cos \frac{\theta }{2}. \end{aligned}$$

The selection of $+$ and − depends on the values of $\omega$, *n* and $\theta _0$. Table 1 shows the selection of positive and negative signs of $N_r$, $N_\varphi$, $f_r$ and $f_\varphi$ in different value ranges of $\omega$, *n* and $\theta _0$. In the planar polar coordinate system, we specify that the directions along the vector ${\varvec{r}}$ and perpendicular to ${\varvec{r}}$ (the direction of $\theta$ increase) are positive. The positive and negative signs in Table 1 indicate whether the components of $N_1$ and *f* are consistent with the positive directions or opposite.

**Table 1:** The selection of positive and negative signs of $N_r$, $N_\varphi$, $f_r$ and $f_\varphi$ in Eqs. (7)–(10) with different ranges of $\omega$, *n* and $\theta _0$.

$\omega$	$>0$				$<0$			
*n*	2*k*		$2k+1$		2*k*		$2k+1$	
$\theta _0$	$(0,\pi )$ $(-\pi ,0)$	$(\pi ,2\pi )$ $(-2\pi ,-\pi )$	$(0,\pi )$ $(-\pi ,0)$	$(\pi ,2\pi )$ $(-2\pi ,-\pi )$	$(0,\pi )$ $(-\pi ,0)$	$(\pi ,2\pi )$ $(-2\pi ,-\pi )$	$(0,\pi )$ $(-\pi ,0)$	$(\pi ,2\pi )$ $(-2\pi ,-\pi )$
$N_r$	−	$+$	$+$	−	−	$+$	$+$	−
$N_\varphi$	−	$+$	$+$	−	−	$+$	$+$	−
$f_r$	$+$	−	−	$+$	−	$+$	$+$	−
$f_\varphi$	−	$+$	$+$	−	$+$	−	−	$+$

During the movement of the bead, according to the definition of work in a planar polar coordinate system^1^, the work done by the radial and angular components of the constraint force $N_1$ and friction *f* is

11 $$\begin{aligned} W_{N_r}= & {} \int {N_r\textrm{d}r}, \end{aligned}$$

12 $$\begin{aligned} W_{N_\varphi }= & {} \int {N_\varphi {r}\textrm{d}\varphi }, \end{aligned}$$

13 $$\begin{aligned} W_{f_r}= & {} \int {f_r\textrm{d}r}, \end{aligned}$$

14 $$\begin{aligned} W_{f_\varphi }= & {} \int {f_\varphi {r}\textrm{d}\varphi }. \end{aligned}$$

The total work done by the combined external force in the process of the bead motion is

15 $$\begin{aligned} W=W_{N_r}+W_{N_\varphi }+W_{f_r}+W_{f_\varphi }. \end{aligned}$$

The kinetic energy of the bead is

16 $$\begin{aligned} E_k=\frac{1}{2}m\left( {\dot{r}}^2+r^2{\dot{\varphi }}^2\right) . \end{aligned}$$

According to the kinetic energy theorem, the kinetic energy of the bead can also be expressed as

17 $$\begin{aligned} E_k=\frac{1}{2}m{v_0}^2+W, \end{aligned}$$

where $v_0$ is the initial velocity of the bead.

## Numerical results

### Analysis of results

This paper uses international units. The units of length, time, mass and angular velocity are $\textrm{m}$, $\textrm{s}$, $\textrm{kg}$ and $\mathrm {rad/s}$ respectively. In the calculation, we let $g=9.8$, $R=1$, $m=1$, $\beta =0$, $\Omega =\Omega _0=2$, $\varphi _0=0$ and time step $\Delta t=10^{-5}$.

**Figure 2:**
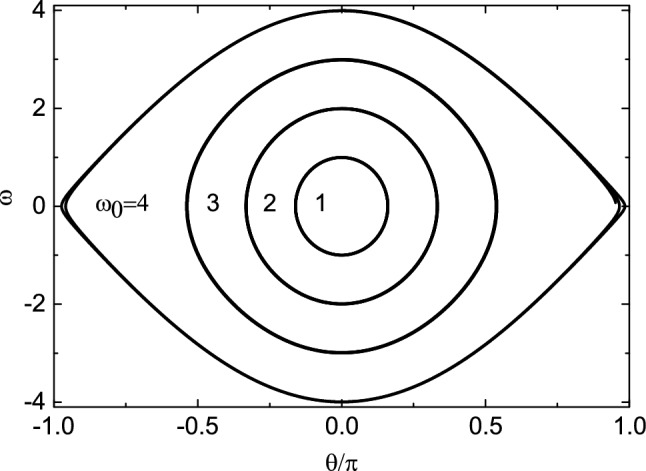
The relationship between $\omega$ and $\theta$ for $\mu =0$. With the increase of the initial angular velocity $\omega _0$, the oscillation range of the bead gradually increases, and the critical angular velocity of the bead oscillating on the hoop is $\omega _c=4$.

In Fig. 2, we display a phase diagram of the relationship between $\theta$ and $\omega$ for $\mu =0$ and $\omega _0=1$, 2, 3 and 4. As can be seen from the figure, the initial angular velocity $\omega _0$ of the bead is too small to rotate around the hoop. Instead, it rotates with the hoop and oscillates periodically near the equilibrium position $O^{'}$ relative to the hoop. The orbits are closed, and the larger the area surrounded by the orbit, the longer the oscillation period. The angular component $N_\varphi$ of the constraint force $N_1$ provides the restoring force. The amplitude of the bead increases with the increase of $\omega _0$, and the maximum oscillation range is $\theta \in (-\pi ,\pi )$. When the initial angular velocity continues to increase, the bead will rotate around the hoop. The critical angular velocity is $\omega _c=4$. This can be explained by the kinetic energy theorem. When the bead moves from $\theta =0$ to $\pi$, if the initial kinetic energy $E_{k0}$ of the bead and the work done by the constraint satisfy $E_{k0}+W_{N_r}+W_{N_\varphi }=0$, the critical initial angular velocity $\omega _c$ can be obtained. If $\omega _0>\omega _c$, then the bead can rotate around the hoop, otherwise the bead oscillates within the range of $\theta \in (-\pi ,\pi )$.

**Figure 3:**
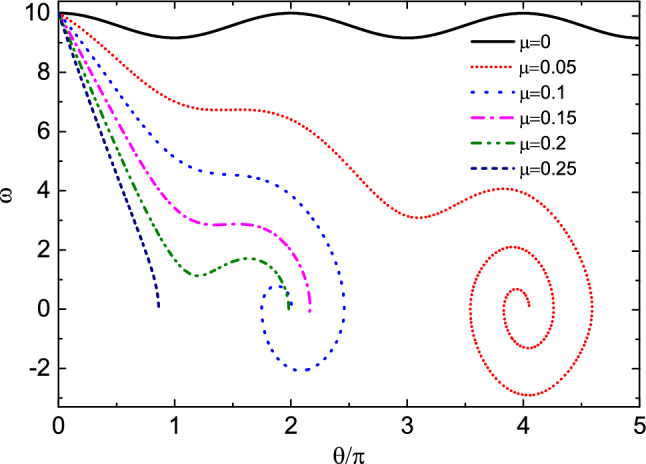
The relationship between $\theta$ and $\omega$ at the initial angular velocity $\omega _0=10$. When $\mu =0$, the bead rotates on the hoop, and the angular velocity changes periodically. With the increase of friction coefficient, the attenuation degree of angular velocity of the bead increases. When $\mu =0.05$, 0.1, 0.15 and 0.2, the bead stops near the equilibrium position $O^{'}$. When $\mu =0.25$, the bead stops near the coordinate origin *O*.

Next, we increase the initial angular velocity $\omega _0$ to study the influence of friction coefficient on the motion of the bead. Figure 3 shows the relationship between $\theta$ and $\omega$ at the initial angular velocity $\omega _0=10$. Table 2 shows the numerical results of the evolution of $\theta$ and $\omega$ with time *t*, and the time interval is 0.5. It can be seen from the figure that when $\mu =0$, the bead rotates on the hoop, and the angular velocity changes periodically relative to $\theta$. When $\mu =0.05$, the kinetic energy of the bead decreases due to the work done by friction. The bead rotates two laps around the hoop, and then does damping oscillation near the equilibrium position $O^{'}$. Finally, the bead stops at the equilibrium position and does circular motion with the hoop. When $\mu =0.1$, 0.15 and 0.2, due to the work done by friction, the kinetic energy loss of the bead is greater, the bead only rotates one lap on the hoop, and the oscillation times at the equilibrium position also decreases or disappears. When $\mu =0.25$, the bead is stationary relative to the hoop after only half a lap around the hoop. At this time, the bead does not stop near the initial equilibrium position $O^{'}$, but near the origin of coordinates *O*. At the coordinate origin, the restoring force $N_\varphi$ of the bead is also 0, but compared to point $O^{'}$, point *O* is less stable, so the probability of the bead stopping at point $O^{'}$ is greater than point *O*. When $\dot{\theta }=0$ and $\dot{\omega }=0$, the fixed point of the system, i.e. the equilibrium position, can be obtained. At this point $\omega =0$. Therefore, from equation Eq. (3a), it can be obtained that

18 $$\begin{aligned} {\Omega _0}^4{\sin ^2\theta }-\frac{\mu ^2g^2}{R^2}-4\mu ^2{\Omega _0}^4{\cos ^4\frac{\theta }{2}}=0. \end{aligned}$$

**Figure 4:**
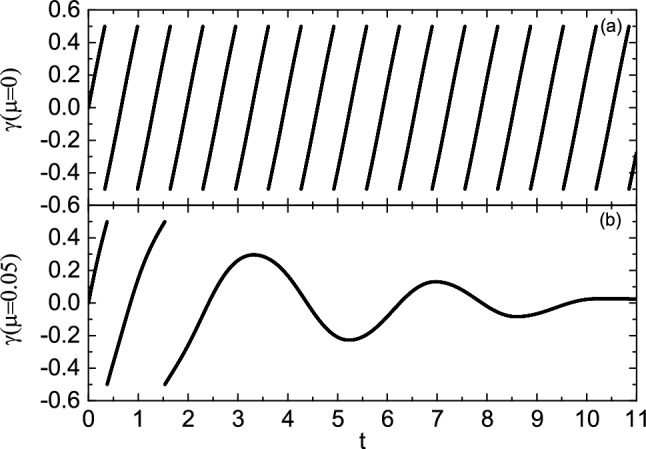
(**a**) and (**b**) are the change of angle $\gamma$ with time t at $\mu =0$ and 0.05, respectively. When $\mu =0$, the bead rotates on the hoop, and the variation range of angle $\gamma$ is $(-\pi /2,\pi /2)$. When $\mu =0.05$, the angular velocity of the bead decreases with time *t*, and the angle $\gamma$ changes continuously, and finally tends to 0.

For a given value of $\mu$, multiple solutions of $\theta$ can be obtained by solving Eq. (18). These solutions are located near points $O^{'}$and *O*. When the values of $\mu$ are 0.05, 0.1, 0.15, 0.20 and 0.25, respectively, it can be seen from Fig. 3 that the values of the stop position $\theta$ of the bead are 4.0494, 2.0092, 2.1357, 1.9824, and 0.8623, respectively. According to solutions of Eq. (18), the fixed point values that are close to the $\theta$ values mentioned above are 4.0504, 1.8986, 1.8465, 1.7922 and 0.7892, respectively. It can be seen that for a relatively small $\mu$ value (e. g. $\mu =0.05$), the bead almost stops at the fixed point. When $\mu$ increases, the friction makes the bead stops near the fixed point. When $\mu =0.15$, 0.2 and 0.25, there is no oscillation process of the bead because its angular velocity has become 0 when it passes near the fixed point, and the larger friction hinders its oscillation. Figure 4 shows the change of angle $\gamma$ with time *t* at $\mu =0$ and 0.05. It can be seen from the figure that when $\mu =0$, the bead rotates on the hoop, the variation range of angle $\gamma$ is $(-\pi /2,\pi /2)$, and there is an angle mutation from $\pi /2$ to $-\pi /2$. When $\mu =0.05$, the bead oscillates near the equilibrium position $O^{'}$ after rotating two laps on the hoop. At this time, the angle $\gamma$ changes continuously, and the variation range of the angle $\gamma$ decreases with the increase of time, and finally tends to 0.

**Table 2:**
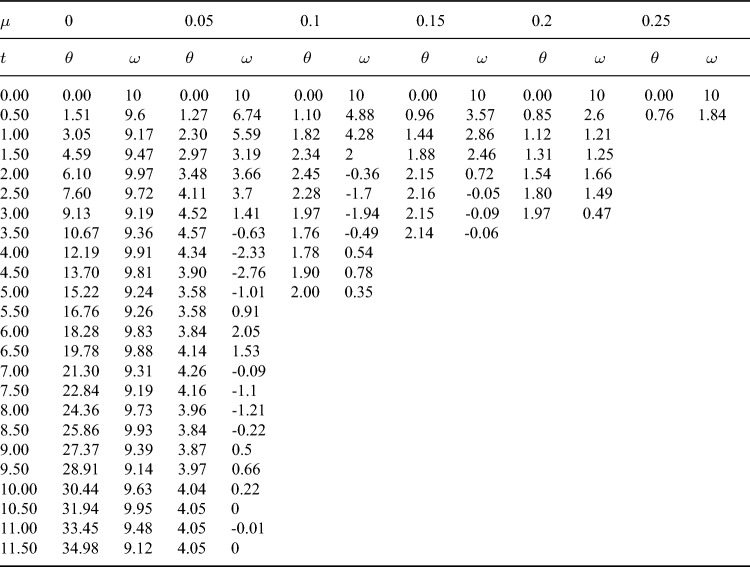
The numerical results of the evolution of $\theta$ and $\omega$ with time *t* in Fig. 3 when $\mu$ =0, 0.05, 0.1, 0.15, 0.2 and 0.25, with a time interval of 0.5.

**Figure 5:**
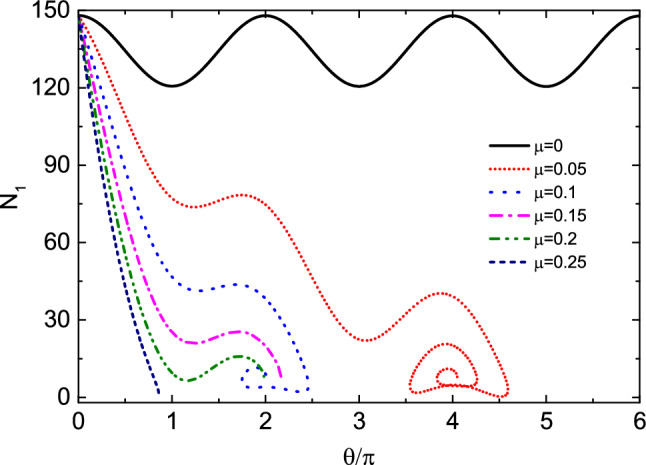
The relationship between the constraint force $N_1$ of the bead and the angle $\theta$ under different friction coefficients $\mu$. The constraint force changes periodically with angle $\theta$ at $\mu =0$. With the increase of $\mu$, the constraint force tends to decrease, and its maximum and minimum values are near the initial position $O^{'}$ and the coordinate origin *O*, respectively.

**Table 3:**
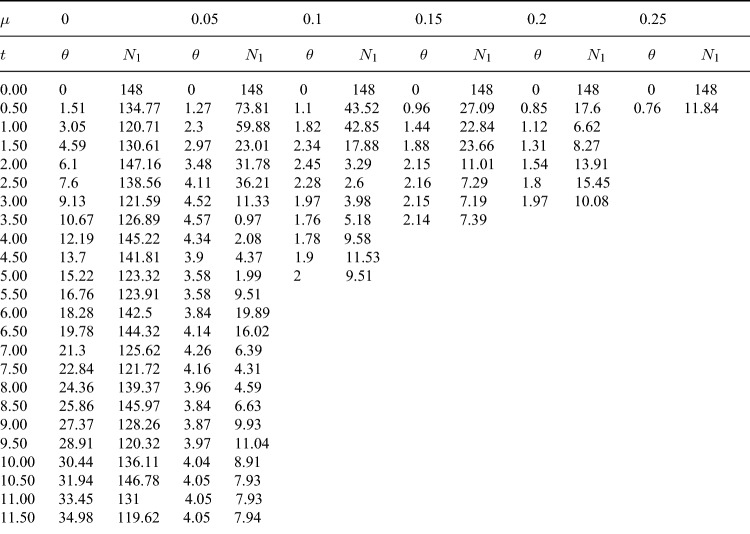
The numerical results of the evolution of $\theta$ and $N_1$ with time *t* in Fig. 5 when $\mu$ =0, 0.05, 0.1, 0.15, 0.2 and 0.25, with a time interval of 0.5.

**Figure 6:**
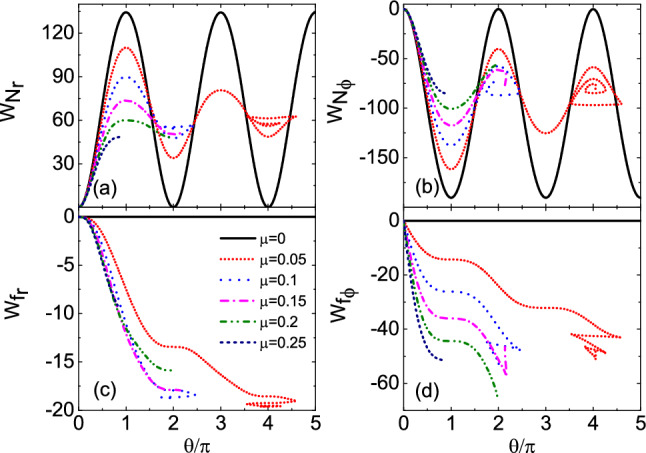
The variation of work done by the radial and angular components of the constraint force $N_1$ and friction *f* with the angle $\theta$ under different friction coefficients $\mu$. The work $W_{N_r}$ and $W_{N_\varphi }$ done by $N_r$ and $N_\varphi$ has a certain periodicity. Radial friction $f_r$ always does negative work and angular friction $f_\varphi$ can do positive work.

**Figure 7:**
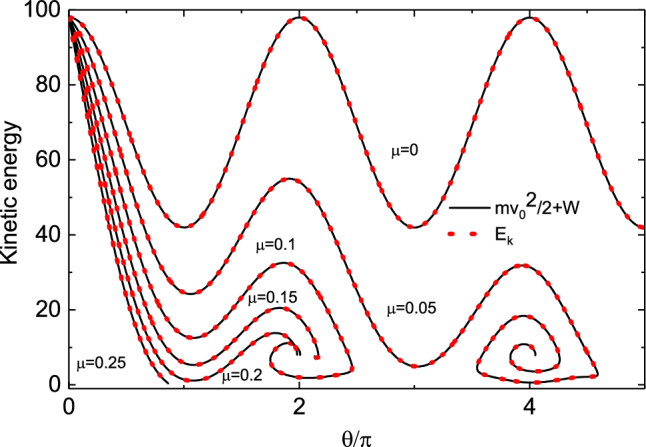
The relationship between kinetic energy and $\theta$ of the bead under different friction coefficients $\mu$. The dotted line and solid line are the calculation results on the left and right of Eq. (17) respectively. It can be seen from the figure that the two calculation results agree well and satisfy the kinetic energy theorem. This also ensures the accuracy of our calculation results.

**Figure 8:**
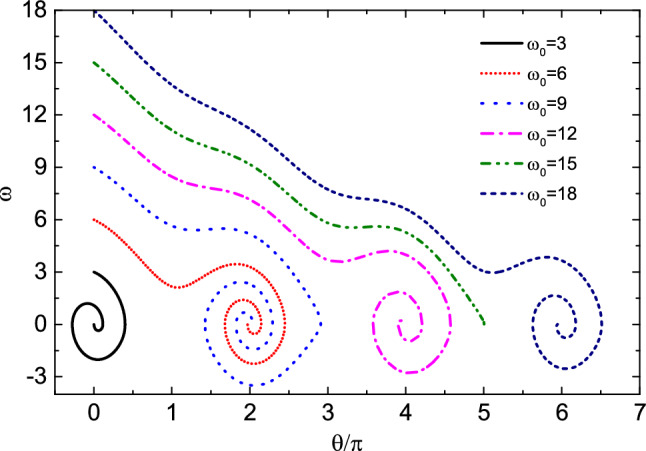
The relationship between $\omega$ and $\theta$ at different initial angular velocities when friction coefficient $\mu =0.06$. When $\omega _0=12$ and 18, the bead rotates 2 and 3 laps around the hoop respectively. When $\omega _0=6$ and 9 the bead rotates one lap on the hoop and then stops at $O^{'}$ point. When $\omega _0=3$, the bead oscillates only near the equilibrium position and then stops at point $O^{'}$. When $\omega _0=15$, the bead rotates 2.5 laps around the hoop and then stops at the origin *O*.

Figure 5 shows the relationship between the constraint force $N_1$ of the bead and the angle $\theta$ under different friction coefficients $\mu$ ($\mu =0$, 0.05, 0.1, 0.15, 0.2 and 0.25). Table 3 shows the numerical results of the evolution of $\theta$ and $N_1$ with time *t*, and the time interval is 0.5. As can be seen from Fig. 5, the constraint force changes periodically with angle $\theta$ at $\mu =0$. With the increase of $\mu$, the constraint force tends to decrease, and its maximum and minimum values are near the initial position $O^{'}$ and the coordinate origin *O*, respectively. This is consistent with the maximum and minimum positions of angular velocity in Fig. 3. Figure 6 shows the variation of work $W_{N_r}$, $W_{N_\varphi }$, $W_{f_r}$ and $W_{f_\varphi }$ done by the radial and angular components of the constraint force $N_1$ and friction *f* with the angle $\theta$ under different friction coefficients $\mu$. As can be seen from Fig. 6a–d, the work $W_{N_r}$ and $W_{N_\varphi }$ done by $N_r$ and $N_\varphi$ has a certain periodicity with the increase of $\theta$. Radial friction $f_r$ always does negative work and angular friction $f_\varphi$ can do positive work. Table 4 shows the positive and negative signs of the work $W_{N_r}$, $W_{N_\varphi }$, $W_{f_r}$ and $W_{f_\varphi }$ done by $N_r$, $N_\varphi$, $f_r$, and $f_\varphi$ with different ranges of $\theta _0$, $\omega$, and $\Omega _0+\beta t+\omega$. In the table, $+$ and − represent positive work and negative work, respectively, which is consistent with the calculation results in Fig. 6.

**Table 4:** Positive and negative signs of the work $W_{N_r}$, $W_{N_\varphi }$, $W_{f_r}$ and $W_{f_\varphi }$ done by $N_r$, $N_\varphi$, $f_r$, and $f_\varphi$ with different ranges of $\theta _0$, $\omega$, and $\Omega _0+\beta t+\omega$. $+$ and − in the table represent positive work and negative work respectively.

$\theta _0$	$(0,\pi )$				$(\pi ,2\pi )$			
$\omega$	$>0$	$>0$	$<0$	$<0$	$>0$	$>0$	$<0$	$<0$
$\Omega _0+\beta t+\omega$	$>0$	$<0$	$>0$	$<0$	$>0$	$<0$	$>0$	$<0$
$W_{N_r}$	$+$	$+$	−	−	−	−	$+$	$+$
$W_{N_\varphi }$	−	$+$	−	$+$	$+$	−	$+$	−
$W_{f_r}$	−	−	−	−	−	−	−	−
$W_{f_\varphi }$	−	$+$	$+$	−	−	$+$	$+$	−

Figure 7 shows the relationship between kinetic energy and $\theta$ of the bead under different friction coefficients $\mu$. We calculated the left and right sides of Eq. (17) respectively. It can be seen from the figure that the two calculation results agree well and satisfy the kinetic energy theorem. This also ensures the accuracy of our calculation results. When $\mu =0$, the kinetic energy of the bead changes periodically with the increase of $\theta$. When $\mu >0$, its kinetic energy decays, and the degree of attenuation increases with the increase of $\mu$.

**Table 5:**
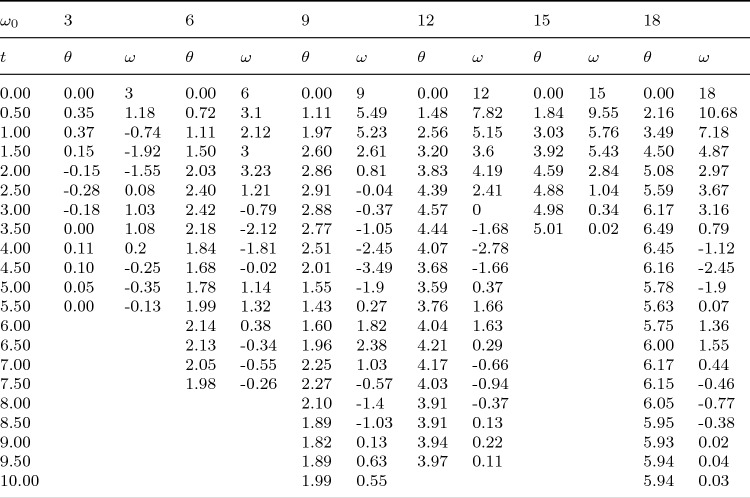
The numerical results of the evolution of $\theta$ and $\omega$ with time *t* in Fig. 8 when $\omega _0$ =3, 6, 9, 12 ,15 and 18, with a time interval of 0.5.

Finally, we study the influence mechanism of initial angular velocity on the kinematics of the bead. Figure 8 shows the relationship between the angular velocity $\omega$ and $\theta$ of the bead at different initial angular velocities $\omega _0$ when friction coefficient $\mu =0.06$. Table 5 shows the numerical results of the evolution of $\theta$ and $\omega$ with time *t*, and the time interval is 0.5. As can be seen from the figure, when $\omega _0=12$ and 18, the bead rotates 2 and 3 laps around the hoop respectively; when $\omega _0=6$ and 9, the bead rotates one lap on the hoop, then does damping oscillation near the equilibrium position and finally stands still relative to the hoop; when $\omega _0=3$, the bead oscillates only near the equilibrium position and then stops at point $O^{'}$. The number of rotation laps of the bead around the hoop is related to the initial angular velocity, but the final oscillation times are basically the same. When $\omega _0=15$, the angular velocity of the bead becomes 0 after rotating 2.5 laps around the hoop. At this time, the bead does not stop at $O^{'}$ point, but near its other fixed point *O*.

### Discussion of results

Next, we will further analyze the types of fixed points. Let the coordinates of the fixed point of the system be $\left( \theta _f,\omega _f\right)$. When $g{\gg }a\left( \omega +\Omega _0\right) ^2+a{\Omega _0}^2\cos \theta$, this represents the situation where the bead is not subject to friction and has a small angular velocity. The Jacobian matrix of the system is

19 $$\begin{aligned} J=\begin{pmatrix} 0&{}-{\Omega _0}^2\cos \theta _f\\ 1&{}0 \end{pmatrix}. \end{aligned}$$

Its intrinsic equation is

20 $$\begin{aligned} {\lambda }^2+{\Omega _0}^2{\cos {\theta _f}}=0. \end{aligned}$$

The coefficient before the first power of $\lambda$ is $\eta =0$. Its eigenvalues are $\lambda _{1,2}={\pm }\Omega _0\sqrt{-\cos {\theta _f}}$. So the fixed point is the center point. The phase orbit forms a closed curve around the fixed point^11^. This is consistent with Fig. 2. When $g{\ll }a\left( \omega +\Omega _0\right) ^2+a{\Omega _0}^2\cos \theta$, this represents the situation where the bead moves at a large angular velocity under the action of friction. The Jacobian matrix of the system is

21 $$\begin{aligned} J=\begin{pmatrix} {\pm }2\mu \Omega _0&{}{\mp }\mu {\Omega _0}^2\sin \theta _f-{\Omega _0}^2\cos \theta _f\\ 1&{}0 \end{pmatrix}. \end{aligned}$$

Its intrinsic equation is

22 $$\begin{aligned} {\lambda }^2{\mp }2\mu \Omega _0\lambda {\pm }\mu {\Omega _0}^2{\sin \theta _f}+{\Omega _0}^2\cos \theta _f=0. \end{aligned}$$

Its eigenvalues are $\lambda _{1,2,3,4}={\pm }\mu \Omega _0{\pm }\Omega _0\sqrt{\mu ^2-\cos \theta _f{\mp }\mu \sin \theta _f}$, $\Delta _{1,2}=4{\Omega _0}^2(\mu ^2-\cos \theta _f{\pm }\mu \sin \theta _f)$. The coefficient before the first power of $\lambda$ is $\eta ={\pm }\mu \Omega _0$. When $\eta =\mu \Omega _0$, it is the case of motion enhancement, and when $\eta =-\mu \Omega _0$, it is the case of motion attenuation. Therefore, the latter is consistent with our model. When $\mu =0.25$, it can be obtained from the fixed point $\theta _f=0.7892$, $\Delta _2=1.0049\>{0}$, $\lambda _3=1.5049\>{0}$, and $\lambda _4=-2.5049<{0}$. This indicates that there is an unstable direction near the fixed point, and the fixed point is a saddle point. When $\mu$=0.05, 0.1, 0.15 and 0.2, $\Delta <{0}$, the fixed point is the stable focus, and the orbit shrinks to the fixed point while rotating^11^. This is consistent with Fig. 3. Through similar analysis, it can be seen that in Fig. 8, when $\theta _f\approx 2n\pi$, the corresponding fixed points are the stable focuses, and when $\theta _f\approx (2n+1)\pi$, the corresponding fixed point are the saddle points.

## Conclusions

In this paper, the orbit constraint of a bead on a rotating large circular hoop in a horizontal plane have been studied. The model we have studied is characterized by considering the existence of friction, which makes the problem more complicated. The dynamic coupling equations of the bead have been derived by using the classical mechanics theory in the polar coordinate system. By numerically solving the coupled equations, the relationship between the angular position of the bead and angular velocity, constraint force, friction and work have been studied. Firstly, we have studied the oscillation of the bead on the hoop without friction. The calculations indicate that the angular component of the constraint force is used as the restoring force to make the bead oscillate periodically on the hoop, and its amplitude increases with the increase of the initial angular velocity. Secondly, we have studied the influence mechanism of friction coefficient on the motion of the bead under a given initial angular velocity. The calculations indicate that the bead will rotate on the hoop, and the number of rotation laps is affected by friction coefficient. When the bead cannot bypass the hoop due to energy loss, it may oscillate near the equilibrium position, and the distance or times of oscillation will decrease with the increase of friction coefficient. Thirdly, we have studied the influence mechanism of different initial angular velocities on the motion of the bead under a given friction coefficient. The calculations indicate that the number of rotation laps of the bead on the hoop is related to the initial angular velocity, but the final oscillation times are basically the same. The bead finally stops at the equilibrium position or coordinate origin. Through the discussion of the results, we found that when the friction is zero, the eigenvalues of the Jacobian matrix of the system are pure imaginary numbers, the fixed point is stable, and the phase diagram is a closed orbit around the stable point. The bead oscillates on the hoop near the fixed point. When the friction is not zero and the eigenvalues are real, the fixed point is an unstable saddle point. The bead circled half a circle on the hoop. When the friction is not zero and the eigenvalues are complex, the fixed points are stable, and the bead has both a circular motion around the hoop and an oscillation near the fixed point. The model in this paper can be used to guide the development of relevant experimental equipment and study the oscillation problem of bead with friction. In addition, the outdoor rotary lifting aircraft in the amusement park can also be approximated as this model, which can be used to qualitatively analyze the force situation of people on the seat.

## Data Availability

Code available on request to corresponding author.

## List of symbols

$$\beta$$: Angular acceleration, $$[rad/s^2]$$
$\gamma$: Included angle, [*rad*]
$$\mu$$: Friction coefficient
$\Omega$: Angular velocity, [*rad*/*s*]
$\omega$: Angular velocity, [*rad*/*s*]
$\Omega _0$: Initial angular velocity, [*rad*/*s*]
$\omega _c$: Critical angular velocity, [*rad*/*s*]
$\theta$: Angular position of the bead on the hoop, [*rad*]
$\varphi$: Polar angle, [*rad*]
$\varphi _0$: Initial angle, [*rad*]
$E_k$: Kinetic energy of the bead, [*J*]
$E_{k0}$: Initial kinetic energy of the bead, [*J*]
*f*: Friction force,[*N*]
$f_\varphi$: Angular component of the friction force, [*N*]
$f_r$: Normal component of the friction force, [*N*]
*g*: Gravitational acceleration, $$[m/s^2]$$
*m*: Mass, [*kg*]
$$N_1$$: Constraint force, [*N*]
$N_2$: Vertical supporting force, [*N*]
$N_\varphi$: Angular component of the constraint force, [*N*]
$N_r$: Normal component of the constraint force, [*N*]
*R*: Radius, [*m*]
*r*: Polar radius, [*m*]
*t*: Time, [*s*]
$$v_0$$: Initial velocity, [*m*/*s*]
*W*: Total work, [*J*]
$W_{f_\varphi }$: Work done by the angular component of the friction, [*J*]
$W_{f_r}$: Work done by the radial component of the friction, [*J*]
$W_{N_\varphi }$: Work done by the angular component of the constraint force, [*J*]
$W_{N_r}$: Work done by the radial component of the constraint force, [*J*]
